# Primary plasma cell leukaemia in a 22-year-old woman: A case report

**DOI:** 10.4102/ajlm.v4i1.289

**Published:** 2015-11-23

**Authors:** Robyn Marshall, Jenifer Vaughan, Ria David, Elise Schapkaitz, Sergio Carmona, Tracey Wiggill

**Affiliations:** 1 Department of Molecular Medicine and Haematology, University of the Witwatersrand, Johannesburg, South Africa; 2 National Health Laboratory Service, Johannesburg, South Africa; 3 Division of Medical Oncology, Department of Internal Medicine, University of the Witwatersrand, Johannesburg, South Africa

## Abstract

**Introduction:**

Primary plasma cell leukaemia is a rare and highly aggressive disease that is commonly diagnosed a decade earlier than multiple myeloma, at a median age of 55 years. However, it has also been described in younger patients, as documented in this case report. It often presents with hepatosplenomegaly and lymphadenopathy, whilst the presence of bony lesions are less-commonly seen when compared to multiple myeloma.

**Case presentation:**

This report describes the case of a young woman who presented with symptoms of anaemia and a history of menorrhagia. On further careful examination, she was found to have additional signs and symptoms and was later diagnosed with primary plasma cell leukaemia.

**Management and outcome:**

On admission, the patient received supportive care measures, including blood products. At diagnosis, a specific chemotherapy regimen was commenced; however, this failed to induce remission. The decision to continue with supportive care only was made and the patient died seven months later.

**Discussion:**

This case study is presented because of its rarity, the young age of the patient at presentation and the unusual clinical and laboratory findings. Persistent anaemia unresponsive to standard treatment should raise the index of suspicion and further investigations directed to exclude malignancies should be considered.

## Introduction

Plasma cell leukaemia (PCL) is a rare and aggressive plasma cell dyscrasia,^1^ which is divided into primary and secondary subtypes. The distinction from multiple myeloma (MM) and other plasma cell dyscrasia is based on the presence of a circulating peripheral blood plasma cell count of > 20% or > 2 × 10^9^/L.^2,3^ Primary PCL presents *de novo* in the leukaemic phase and comprises 60% of all PCL.^3,4^ It has a median age of diagnosis of 55 years, a decade earlier than MM and secondary PCL, with the symptoms often mimicking those of acute leukaemia.^5^

Prognostic parameters include a low serum albumin; hypercalcaemia and elevated β_2 _microglobulin, serum lactate dehydrogenase and serum C-reactive protein; an absolute peripheral blood plasma cell count of > 4 × 10^9^/L; thrombocytopenia and an increased percentage of S-phase plasma cells; advanced age (age at diagnosis of 60 years or older) and poor performance status (Eastern Cooperative Oncology Group grade of ≥ 2),^6,7^ based on a patient's ability to perform the normal activities of daily living. Specific cytogenetic results associated with lower overall survival rates include a complex karyotype, hypodiploidy, as well as several deletions and translocations.^6^

## Ethical considerations

Consent was obtained from the patient with ethical clearance from the University of the Witwatersrand Human Research Ethics Committee. The ethics clearance number is M130269.

### Potential benefits and hazards

There were no risks to the subject involved in this case report; and no potential physical or psychological dangers were anticipated. There was no perceived benefit to this patient. No information that could identify the patient has been published and the authors have endeavoured to maintain the patient's anonymity. It is hoped that other patients with a similar presentation of severe anaemia and a serious underlying condition may be clinically managed more quickly.

### Recruitment procedures

As this was a case study that highlighted a rare and interesting case, the patient was requested to provide consent to make use of her clinical information, including examination findings, special investigation results and treatment strategies applied**.** Entitlement to withdraw consent would lapse once the case report was submitted for publication.

### Informed consent

The patient was requested to provide written consent to allow for all or any part of this material collected (other than unique patient identifiers) to appear as an abstract, a case study, or an article in a journal, and any other works or products, in any form or medium.

### Data protection

The patient's name has not been published with the material and the authors have endeavoured to assure anonymity. However, it is understood that despite the best efforts of the authors, the possibility that someone, for example, members of the patient's family or the healthcare staff, may recognise the patient from the images and/or the accompanying text. All data collected for this case report was available only to the authors of the case report and no other party had access to any of the patient's individual identifiers.

## Case presentation

**FIGURE 1:**
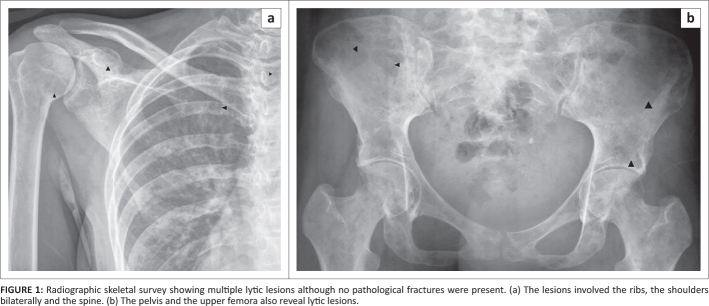
Radiographic skeletal survey showing multiple lytic lesions although no pathological fractures were present. (a) The lesions involved the ribs, the shoulders bilaterally and the spine. (b) The pelvis and the upper femora also reveal lytic lesions.

A 22-year-old female patient presented at a peripheral clinic in September 2012 with a history of heavy menses after receiving medroxyprogesterone acetate for contraceptive purposes in July 2012. At this time she complained of fatigue, dizziness, lower back pain and poor appetite; iron supplements were prescribed. In November 2012 she presented at a local hospital complaining of severe back pain and persistent vaginal bleeding. Her past medical history revealed that this had been ongoing for more than one month. On admission she was found to have no significant lymphadenopathy or hepatosplenomegaly. She was also tachycardic and an echocardiogram revealed a functional ejection systolic murmur. A radiographic skeletal survey showed multiple lytic lesions (Figure 1). All pertinent laboratory investigations are summarised in Table 1.

**FIGURE 2:**
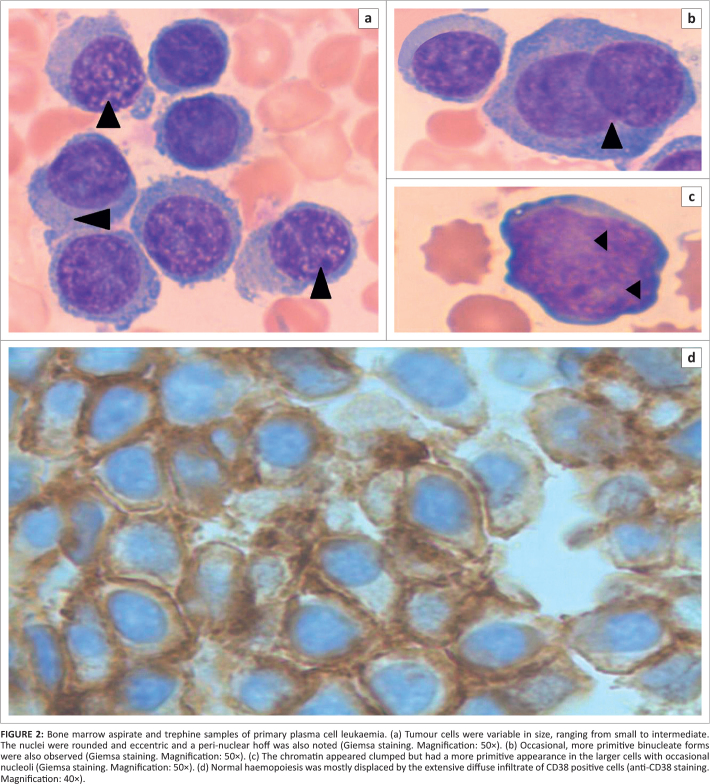
Bone marrow aspirate and trephine samples of primary plasma cell leukaemia. (a) Tumour cells were variable in size, ranging from small to intermediate. The nuclei were rounded and eccentric and a peri-nuclear hoff was also noted (Giemsa staining. Magnification: 50×). (b) Occasional, more primitive binucleate forms were also observed (Giemsa staining. Magnification: 50×). (c) The chromatin appeared clumped but had a more primitive appearance in the larger cells with occasional nucleoli (Giemsa staining. Magnification: 50×). (d) Normal haemopoiesis was mostly displaced by the extensive diffuse infiltrate of CD38 positive cells (anti-CD38 staining. Magnification: 40×).

A number of baseline and definitive special investigations were performed (Table 1). A bone marrow investigation revealed hypercellular marrow with extensive infiltration by a population of abnormal plasma cells similar to that described in the peripheral blood (Figure 2A, 2B and 2C). The infiltrate was positive for immunohistochemical stains, including CD38 (Figure 2D), CD56 and MUM-1, but was negative for cyclin-D1, CD20 and CD45. Fewer than 20% of cells were positive for Ki-67. Immunophenotypic analysis revealed a population of ~60% – 70% large, more complex cells that expressed bright CD38, moderate CD138 and bright aberrant CD56 with dim kappa light-chain restriction.

Fluorescence in situ hybridisation analysis revealed that the cells were positive for a deletion at 13q14.3, showed trisomy of chromosome 18 and loss of the fibroblast growth factor receptor (*FGFR3)* gene. Chromosomal analysis of the bone marrow cultures revealed a complex karyotype (Table 1). Features of note were numerical aberrations, including monosomy of chromosomes 12, 13 and 14 and trisomy of chromosome 18. Structural aberrations included partial deletion of chromosome 1p and an extra chromosome 1 with a partial deletion on the p-arm, leading to an additional copy of the 1q region. There was a translocation involving chromosomes 3 and 14, leading to rearrangement of the immunoglobulin heavy chain locus (*IgH@*) gene. Terminal deletion of chromosome 4p confirmed the deletion of the *FGFR3* gene.

**TABLE 1:** Summary of relevant laboratory investigations performed on this patient.

Investigation (units)	Patient value (normal range)
**Full blood count**	
WCC (10^9^/L)	44.9 (4.00–10.00)
Hb (g/dL)	3.9 (12.1–16.3)
MCV (fl)	89.5 (79.1–98.9)
Plt (10^9^/L)	39 (178–400)
Other (plasma) cells (10^9^/L)	33.68 (75%)
**Biochemistry**	
LDH (U/L)	1332 (208–378)
Corrected Ca++ (mmol/L)	3.17 (2.15–2.5)
β_2_microglobulin (mg/L)	3.4 (0.7–1.8)
**Immunoelectrophoresis**	
Urinary Bence Jones Proteins (g/L)	0.47 Kappa light chains
**Serum protein electrophoresis**	
Monoclonal band (g/L)	0.72
	Free kappa light chains
	No heavy chains: IgG, IgM, IgD, IgE
	Immune paresis
**Serum free light chains (g/L)**	Kappa: 3925; Lambda: < 0.8
**FISH analysis**	
Present	13q14.3 deletion, Trisomy 18, Loss of *FGFR3* gene
Absent	*TP53* deletion, translocation t(4;14), *MYC* break-apart
**Chromosomal analysis**	Complex karyotype:
	44~45,XX,del(1)(p21),+del(1)(p13.2),t(3;14)(q?13;q32), -12,-13,-14,der(16)t(16;?)(q?21;?),+18,der(22)t(22;?)(p11.2;?)[3]/44~45,idem,del(4) (p16)[1]/45~46,idem,del(4)(p16),+mar[7]/45~46,idem,+mar[2]/46,XX[7]

WCC, white cell count; Hb, haemoglobin; MCV, mean cell volume; Plt, platelet count; LDH, lactate dehydrogenase; Ca++, calcium; FISH, fluorescence in situ hybridisation.

## Management and outcome

On admission, the patient was stabilised and given fresh frozen plasma, platelets and packed cells. With confirmation of the diagnosis, the patient was started on a chemotherapy regimen which included: vincristine 3.6 mg/m^2 ^intravenously on days 1–4 (D1–D4), doxorubicin 9 mg/m^2^ intravenously on D1–D4, dexamethasone 40 mg orally on D1–D4, dexamethasone 40 mg orally on D9–D12, dexamethasone 40 mg orally on D17–D20. She received one cycle of therapy; however, this induction failed to induce remission.

## Discussion

*De novo* PCL is a rare disease and presentation in patients aged younger than 40 years is exceptionally rare. To the best of our knowledge, only two case reports of such patients, in whom primary PCL presented at aged 30 and 21 years, have been reported in the literature.^8,9^ As is often reported, this patient presented with symptoms suggestive of an acute leukaemia. However, some of her presenting symptoms would more commonly be seen in secondary PCL and MM, including the presence of bone pain and lytic lesions. There was no evidence of extra-medullary deposits and there was an absence of hepatomegaly, splenomegaly and lymphadenopathy. There was no evidence of a pleural effusion and no renal dysfunction, which are commonly described in primary PCL.^5,7,10,11^ In addition to some of the unusual clinical features noted, atypical laboratory features were also found, including the presence of bright CD56 expression on flow cytometric assessment and the absence of CD20 on immunohistochemical staining.^10^

Cytogenetic abnormalities are a common feature of PCL, with 70% of patients with primary PCL and 100% of patients with secondary PCL presenting with abnormal karyotypes.^5^ A recent study that provided a genomic characterisation of patients with primary PCL revealed a significant overlap with characteristics seen in MM, although *TP53* deletions, complex karyotypes, hypodiploidy and *IgH@* translocations were more frequently present in PCL.^4,12,13^ These *IgH@* translocations were identified in 87% of primary PCL cases, del(13q) in 74% of cases and del(17p) in 35% of cases.^4^ In addition, abnormalities in chromosome 1 are frequent in PCL, particularly 1q21 amplification and del(1p), a deletion that has been associated with shorter overall survival.^14,15^ Both monosomy 13 and trisomy 18 are common in PCL, with monosomy 13 occurring in up to 85% of cases and trisomy 18 in 43%.^7,16^ All of the aberrations described above were part of this patient's karyotype, except for the *TP53* deletions. Monosomy 12 and 14 have also been reported in primary PCL in the form of case reports.^17^

Poor prognostic factors in primary PCL include both clinical and laboratory parameters.^6,7^ Of these, the case study patient presented with a performance status score of ≥ 2, an absolute peripheral blood plasma cell count of > 4 × 10^9^/L, thrombocytopenia, diffuse marrow infiltration, specific cytogenetic abnormalities, including a complex karyotype, elevated lactate dehydrogenase, elevated β_2 _microglobulin and hypercalcaemia. Induction therapy failed to induce remission.

Survival is known to be poor in this category of plasma cell dyscrasia, with 28% of patients dying in the first month following diagnosis.^10^ The average survival for primary PCL is 11.2 months.^5^ In general, treatment is aimed at improving quality of life and prolonging survival. Therapy initiation should begin promptly and aim for rapid disease control in an attempt to prevent early death.^11^ Chemotherapy regimens have previously been based on those used for MM, with no specific standard protocol available. Of these, intensive multidrug regimens with an alkylating agent as a base have been used with limited success and more recently bortezomib-based regimens are recommended, followed by autologous stem cell transplantation, if feasible.^6,10^ Allogeneic transplantation can be considered in younger patients.^6^

Our patient received one cycle of induction therapy with a modified vincristine, doxorubicin, dexamethasone regimen, as bortezomib therapy was not available. With the regimen provided, she never obtained complete remission and autologous transplant was not possible. She subsequently received palliative care and died in hospital seven months after the initial diagnosis.

This case was presented because of its rarity and the young age of the patient at presentation, as well as its unusual laboratory and clinical features. This was an unexpected diagnosis and, not surprisingly, the diagnosis was not made at first presentation. A history of recent contraceptive use, menorrhagia and a resultant iron deficiency was not an uncommon finding. However, additional symptoms were also noted and the patient had no improvement, despite iron supplementation. A high index of suspicion, together with early referral and the use of basic first line investigations, such as differential count, should be advocated. The inaccessibility of the recommended drug therapy was also likely to have affected survival and diminished chances for possible stem-cell transplant.

It is vital to make an early diagnosis of haematological and other malignancies in our setting where treatment options are already limited and delayed diagnosis may negatively impact prognosis.
